# Larval *Helicoverpa zea* Transcriptional, Growth and Behavioral Responses to Nicotine and *Nicotiana tabacum*

**DOI:** 10.3390/insects5030668

**Published:** 2014-09-12

**Authors:** Linus Gog, Heiko Vogel, Sue M. Hum-Musser, Jason Tuter, Richard O. Musser

**Affiliations:** 1 Department of Biological Sciences, Western Illinois University, Waggoner Hall 358, Macomb, IL 61455, USA; E-Mails: linusgog@uiuc.edu (L.G.); sm-hum-musser@wiu.edu (S.M.H.-M.); je-tuter@wiu.edu (J.T.); 2 Department of Entomology, Max Planck Institute for Chemical Ecology, Beutenberg Campus, Jena 07745, Germany; E-Mail: hvogel@ice.mpg.de

**Keywords:** cannibalism, chemoreceptor, cytochrome P450, generalist, glucose oxidase, herbivory, nicotine

## Abstract

The polyphagous feeding habits of the corn earworm, *Helicoverpa zea* (Boddie), underscore its status as a major agricultural pest with a wide geographic distribution and host plant repertoire. To study the transcriptomic response to toxins in diet, we conducted a microarray analysis of *H. zea* caterpillars feeding on artificial diet, diet laced with nicotine and *Nicotiana tabacum* (L.) plants. We supplemented our analysis with growth and aversion bioassays. The transcriptome reflects an abundant expression of proteases, chitin, cytochrome P450 and immune-related genes, many of which are shared between the two experimental treatments. However, the tobacco treatment tended to elicit stronger transcriptional responses than nicotine-laced diet. The salivary factor glucose oxidase, known to suppress nicotine induction in the plant, was upregulated by *H. zea* in response to tobacco but not to nicotine-laced diet. Reduced caterpillar growth rates accompanied the broad regulation of genes associated with growth, such as juvenile hormone epoxide hydrolase. The differential expression of chemosensory proteins, such as odorant binding-protein-2 precursor, as well as the neurotransmitter nicotinic-acetylcholine-receptor subunit 9, highlights candidate genes regulating aversive behavior towards nicotine. We suggest that an observed coincidental rise in cannibalistic behavior and regulation of proteases and protease inhibitors in *H. zea* larvae signify a compensatory response to induced plant defenses.

## 1. Introduction

*Helicoverpa zea* is an agriculturally important generalist pest on a large number of crop plants. As a highly polyphagous herbivore, the most destructive stage is when the young larvae feed gregariously, while older larvae can become aggressive and cannibalistic [1]. Caterpillars are occupied with growth until pupation and observations on the foraging behavior of insects maintain that herbivores balance their nutrient intake against various constraints, posed in chief by food source limitations, plant chemical defenses [2] and the risk of predation [3]. Conventional distinctions drawn between specialist and generalist approaches to constraints on feeding regard the two strategies as a trade-off between feeding efficiency and the opportunities of choice, whereby specialization confers the benefits of optimized physiology to a narrow selection of food sources, while the capacity and action of choosing among the food options afforded by the generalist approach detracts from feeding efficacy (for discussion, see Singer [4] and Bernays [5]). This distinction is apparent on a genomic level; in a recent microarray comparison of specialist and generalist caterpillar transcriptomes, changes in gene regulation in the specialist *Manduca sexta* directly corresponded to changes in the chemical defense of its host plant, *Nicotiana attenuata*. By contrast, the transcriptomic responses of a generalist caterpillar, *Heliothis virescens*, were broader and less specific to the same changes in defense chemistry of the same host-plant [6]. For a comprehensive review of the transcriptome response of insect herbivores to host plant and toxins, see Vogel *et al*. [7].

The polyphagous habit of *H. zea* caterpillars extends to cannibalism. Although cannibalism is a common occurrence among juvenile lepidopterans, its causes are not always clear [1,8]. One explanation holds that *H. zea* caterpillars engage in cannibalism as a matter of resource competition. Alternatively, Bernays [9] suggests that polyphagous herbivores might compensate for the presence of toxins or nutritional deficiencies in their diet by feeding on conspecifics. Indeed, in a preference assay between Bt-corn (corn expressing *Bacillus thuringiensis* toxin) and non-Bt corn diet, *H. zea* caterpillars were more likely to engage in cannibalism when provided with Bt-corn diet than control diet [10]. In one rare instance of predaceous behavior, an *H. zea* caterpillar eschewed plant material in favor of other caterpillars; however, *H. zea* caterpillars are not usually carnivorous [1].

*Helicoverpa zea* can be a pest of tobacco, *Nicotiana tabacum* (L.). *Nicotiana* plants deter herbivores by producing the toxic alkaloid nicotine [11]. As a lethal neurotoxin, nicotine effectively curtails the rate at which herbivores can ingest plant tissue [12]. *Nicotiana* plants also produce trypsin inhibitors, which prevent herbivores from digesting protein. The sum of nicotine and trypsin inhibitors acting in concert impacts caterpillar growth more than either of the two defense compounds acting alone [12]. Although the combined defense presented by nicotine and trypsin inhibitors is unique to the genus *Nicotiana*, their effect of rendering the plant indigestible is also documented in defensive compounds produced by tomato, *Solanum*, plants [13]. Indigestibility is thought to be a motif common to plant defenses in general [13].

Because the toxicity of nicotine presents a formidable obstacle to growth, understanding how *H. zea* caterpillars cope with tobacco chemical defense may yield further insight on how the corn earworm has gained such a wide geographic distribution and extensive menu of host plants. Because both behavioral and physiological adjustments are essential features of the generalist feeding strategy, we supplemented a transcriptomic profile of sixth-instar *H. zea* caterpillars feeding on nicotine-laced diet and tobacco plants with a series of aversion bioassays. In these bioassays, we presented *H. zea* caterpillars with a choice between two feeding constraints, posed by the toxicity of nicotine against the risk of predation in the form of cannibalism. Overall, we expected differences in diet to be accompanied by differences in both the behavior and transcriptome of *H. zea*.

## 2. Experimental

### 2.1. Artificial Diet Preparation, Insect Rearing and Plant Rearing

Commercially available artificial diet was prepared according to the supplier’s instructions (Southland Products Inc., Lake Village, AR, USA). We modified this recipe to include 5 g of the antibiotic azomycin, for a final concentration of 3 µg/mg diet, to reduce bacterial growth. Experimental diet treatments were made by including nicotine (Sigma-Aldrich^®^, St. Louis, MO, USA) to the artificial diet to a final concentration of 0.75 µg nicotine/mg diet or 1.5 µg nicotine/mg diet.

*Helicoverpa zea* caterpillars were obtained as neonates from the USDA (National Center for Agricultural Utilization Research, Peoria, IL, USA). Once hatched, the neonates allowed to feed *ad libitum* on an excess of artificial diet (~3–4 g) in individual medicine cups (Solo Cup Company, Lake Forest, IL, USA) and incubated in a growth chamber (33 °C, 14 h of light/day). Caterpillars were periodically sorted by instar. Early 6th instar caterpillars ~12 h after molting were removed for experiments.

*Nicotiana tabacum* plants were grown from seed in a growth chamber (30 °C, 14 h of light/day). Seedlings were grown in commercially available potting soil (Sunshine Professional Potting Mix, Sun Gro Horticulture, Vancouver, BC, Canada) in 1 L plastic pots and fertilized once a week (N:P:K = 24:8:16, Expert Gardener All Purpose Water Soluble Plant Food, Chemisco, St. Louis, MO, USA).

### 2.2. Growth Bioassay

*Helicoverpa zea* neonates were weighed and reared on artificial diet and artificial diet laced with low (0.75 µg nicotine/mg diet) and high (1.5 µg nicotine/mg diet) doses of nicotine. Following a 5-day incubation period, *H. zea* caterpillars were removed from the control and nicotine-laced diet. The caterpillars were weighed to measure weight gain. The results were analyzed in R [14] via ANOVA with a Fisher’s LSD *post hoc* test.

### 2.3. Behavioral Assays

*Nicotine aversion bioassay*: Artificial diet (control treatment) and artificial diet laced with nicotine (experimental treatment: 1.5 µg nicotine/mg diet) was measured into individual portions weighing ~2.5 g. Portions of each diet treatment were placed approximately 3 cm apart from one another in Petri dishes. One 6th instar *H. zea* was placed in each Petri dish. Petri dishes were sealed with Parafilm^®^ to prevent moisture loss. *Helicoverpa zea* caterpillars were removed after 28 h of feeding. Caterpillar frass was removed from Petri dishes using a spatula. Control diet and nicotine-laced diet were measured. Measurement data were analyzed via *t*-test in R.

*Incidence of cannibalism experiment*: The growth bioassay was repeated a second time as described, with the exception that one experimental diet treatment (nicotine-laced diet or control diet) was replaced with an additional 6th instar *H. zea* caterpillar. Caterpillar feeding was observed for incidences of cannibalism induced mortality. As before, the amount of diet consumed was weighed and measured. Incidence rates of cannibalism were analyzed via Wilcoxon test in R.

### 2.4. Microarray Experiment

Feeding enclosures were constructed by fastening the open ends of two plastic tubs (54.6 cm × 95.25 cm × 113.8 cm; Newell Rubbermaid, Atlanta, GA, USA) to one another with packing tape (3M, St. Paul, MN, USA). Rectangular holes (22 cm × 42 cm) were cut out of the sides of the top container to allow for air circulation. A glue gun was used to attach clear plastic mesh over the ventilation holes. Five vegetative, six-week old *N. tabacum* plants were placed inside the feeding enclosures. Ten cubes each of ~2.5 g portions of artificial diet and artificial diet laced with nicotine were placed in a smaller clear plastic tub (30.23 cm × 43.69 cm × 36.58 cm; Newell Rubbermaid, Atlanta, GA, USA). Ten 6th instar *H. zea* caterpillars were placed in each of the three feeding enclosures. Two *Helicoverpa zea* caterpillars were initially evenly distributed on the leaves of each of the five *N. tabacum* plants. Caterpillars were fed for 24 h under intermediate observation. Following 24 h, caterpillars were flash frozen in liquid nitrogen and stored at −80 °C until preparation for transcriptomic analysis. An additional experiment but with tobacco plants treated 24 h prior with jasmonic acid (JA, Sigma-Aldrich^®^) at 100 µM in 1% acetone, a plant hormone widely known to stimulate plant defenses such as nicotine, was performed in a similar manner. The control plants were sprayed with only 1% acetone. The leaves were sprayed with designated treatment until run off was observed. These experiments were conducted once with five plants and/or 10 caterpillars per treatment. Individual caterpillars harvested from different plants were biological replicates.

### 2.5. Total RNA Purification and Labeling

Individual caterpillars were ground to a fine powder under liquid nitrogen. Total RNA was extracted from 0.2 mg of each caterpillar sample using TRIzol^®^ reagent (Life Technologies, Invitrogen, Carlsbad, CA, USA), following the manufacturer’s protocol. Total RNA quality was assessed with a Nanodrop 2000 UV-VIS Spectrophotometer (Thermo Fisher Scientific, Inc., Waltham, MA, USA). Total RNA samples exhibiting 260/280 nm absorption ratios exceeding 1.90 qualified for use in microarray analysis.

### 2.6. Amplification and Labeling mRNA

The Agilent Gene Expression Quick-Amp Labeling Cyanine CTP Dye Kit (Agilent Technologies, Santa Clara, CA, USA) was used to amplify total RNA, following the manufacturer’s protocol. Individual samples from each treatment were alternately labeled with either Cy-3 or Cy-5 dye. Labeling efficiency and cRNA concentration was assessed via Nanodrop 2000. Labeling efficiency was defined as specific activity of the cyanide dye, measured as dye pmols/cRNA ng/µL. Samples exhibiting specific activity of 6.0 pmols/ng/µL qualified for microarray hybridization.

### 2.7. Microarray Hybridization

The cRNA was hybridized to *Helicoverpa armigera* microarray slides (Agilent Technologies, Inc., Santa Clara, CA, USA) developed in part by Heiko Vogel (Max Planck Institute for Chemical Ecology, Jena, Germany). Each microarray slide is spotted with four arrays of 44,000 oligonucleotides with approximately 27,000 putative gene objects. The microarray design is described in detail in Celorio-Mancera *et al.* [15]. Microarrays were hybridized with labeled cRNA targets following the Agilent Low Input Quick Amp Labeling protocol as described by Noland *et al.* [16]. Microarrays were hybridized with Cy-3 and Cy-5 labeled cRNA targets and dye reversed for each experimental treatment. We successfully hybridized eight replicates of caterpillars that had fed on control/artificial diet, four replicates of caterpillars that fed on artificial diet coated with nicotine, six replicates of caterpillars that fed on tobacco plants, and three replicates of caterpillars that fed on tobacco plants treated with jasmonate. All of the replicates were biological and not technical replicates consisting of two caterpillars each.

### 2.8. Microarray Scanning and Analysis

Microarrays were scanned with an Axon Instruments GenePix Personal 4100 scanner (Axon Molecular Devices, Foster City, CA, USA). Microarrays were scanned individually at the default laser intensity of the instrument and the photomultiplier tube was set to reduce the number of saturated spots below 10% and scaled between each image manually to overall ratio of nearly 1. Data collected from each experiment were uploaded into the Genesifter^®^ software [17], which was used to log-transform and scale feature intensities across all microarrays. An ANOVA that was multiple test corrected with a Benjamini-Hochberg statistical correction was used to control for family-wise error rate found in microarray studies [18]. Genes with significantly up- or downregulated expression were sorted into functional groups and gene groups based on literature searches. Statistically significant genes were subjected to Correlation Method hierarchical cluster analysis in Genesifter^®^.

### 2.9. Primer Design and qPCR

Real-time quantitative polymerase chain reaction was performed on three treatments: control caterpillars on artificial diet, caterpillars on artificial diet treated with nicotine, and caterpillars feeding on tobacco plants. Four biological replicates using the same RNA samples previously isolated as described above were used to confirm the microarray data. We prepared the cDNA using the Verso cDNA Synthesis Kit (Thermo Fisher Scientific Inc., Waltham, MA, USA) and the qPCR reactions with Fast SYBR Green Master Mix (Applied Biosystems by Life Technologies, Carlsbad, CA, USA) following the methods described in Musser *et al.* [19] and Suzuki *et al.* [20]. Genes of interest were compared against an endogenous control gene, alpha-tubulin, using the Applied Biosystems Step-One Plus qPCR system. Five housekeeping genes (actin A3b, alpha-tubulin, eukaryotic initiation factor 5C, ribosomal protein L13, and glycealdehyde-3-phosphate dehydrogenase) were identified and examined based on the stability of their expression from the microarray and qPCR results, and other studies [15,21]. Alpha-tubulin was selected as the best endogenous control gene. Selected template cDNA primers were targeted close to the 3' end of each gene. The primers used are shown in Table 1. The qPCR delta-delta CT analysis method was used to determine the magnitude of differential regulation for each gene tested between caterpillars on control diet versus caterpillar on nicotine-treated diet or tobacco plants [22].

**Table 1:** Primers designed to verify gene expression.

Gene Identifier^1^	Gene Name	Forward Primer	Reverse Primer
AF286059.1	actin A3b (ACTIN)	5'-gcatccacgagaccacctac-3'	5'-cctccggacagaactgtgtt-3'
FJ997341.1	aminopeptidase (APEP)	5'-gccacgtctaggtccaacat-3'	5'-ggagctgctgtagtggtcgt-3'
JQ069957.1	αTubulin (aTUB)	5'-catgttgtaccgtggagacg-3'	5'-ctggtagttgatgcccacct-3'
AAX62028.1	chymotrypsin (CMT)	5'-gttctcagcaacagcgagtg-3'	5'-aggaggtcacaccaatcagg-3'
FJ493468.1	ecdysone oxidase (EOX)	5'-cgaccacgtaagagtgctga-3'	5'-caacgtgtagcactcccaga-3'
EU629216.1	glucose oxidase (GOX)	5'-tgactgctccaagactggtg-3'	5'-gggtctgtcgagttgatggt-3'
JF417983	glyceraldehyde-3-phosphate dehydrogenase (GADPH)	5'-gaagggcatcctcgactaca-3'	5'-atgacacggttggagtagcc-3'
Har_00110845	juvenile hormone inducible protein (JH)	5'-ggtctatccacgcgatgtct-3'	5'-gaacggaacagcctctaacg-3'
EU325554	lipase (LPS)	5'-catccagtgtacgcaccaac-3'	5'-aaagcccgtagattccgttt-3'
AAX62028.1	trypsin (TRYP)	5'-aacctcttccggtggttctt-3'	5'-ccacgggtagcgtagttgtt-3'
Jq744275.1	ribosomal protein L13 (RPL13)	5'-ccgaagggcaagaaggtatt-3'	5'-attaatggtccacggagctg-3'

^1^ Gene identifier refers to the microarray gene ID or NCBI Accession ID [23].

## 3. Results and Discussion

The results support our initial hypothesis that the transcriptome, growth and behavior of *H.* zea caterpillars would all change with the addition of nicotine to the caterpillar’s diet and to feeding on tobacco plants. This effect is characterized by an observed tendency for the caterpillars to compensate for defenses known to exist in *Nicotiana* plants, notably nicotine and protease inhibitors [12]. The transcriptome of the *H. zea* caterpillars, as documented by the microarray analysis below, reflect numerous physiological responses to nicotine and tobacco that would not otherwise be qualitatively apparent. For instance, genes coding for cytochrome P450 enzymes, known to form the basis for nicotine detoxification in nicotine-tolerant species [24], were both up- and downregulated in our experiment. The data also reveal the differential expression of genes related to immune functions, typically associated with insect defense against bacterial, fungal and viral pathogens.

### 3.1. Growth Bioassay Results

**Figure 1:**
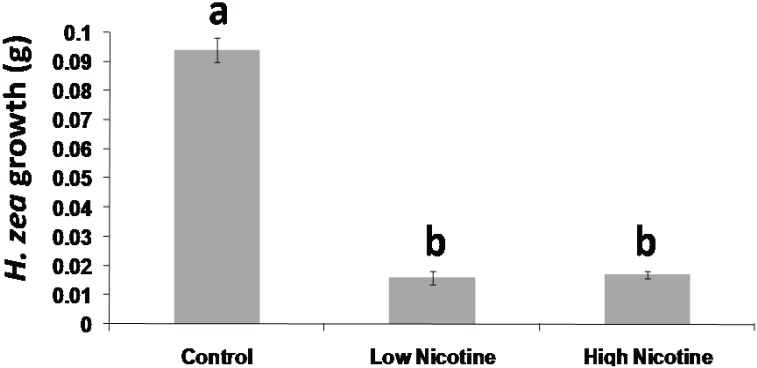
Growth bioassay. *Helicoverpa zea* neonates feeding on artificial control diet gained significantly more mass over a five-day period than neonates feeding on artificial diet laced with Low (0.75 µg nicotine/mg diet) and High (1.5 µg nicotine/mg diet) concentrations of nicotine. (Control N = 72, Low N = 95, High N = 47, Fishers LSD. *p* = 0.0025).

The presence of nicotine at either 0.75 or 1.5 μg nicotine/mg diet reduced neonate growth rate by a factor of 6 (Figure 1) (Fisher’s LSD, *p* < 0.05). The low level of nicotine was the estimated concentration found in tobacco leaves and was enough to have a significant effect on *H. zea* larval growth rates.

### 3.2. Behavioral Assays

#### 3.2.1. Nicotine Aversion Bioassay Results

**Figure 2:**
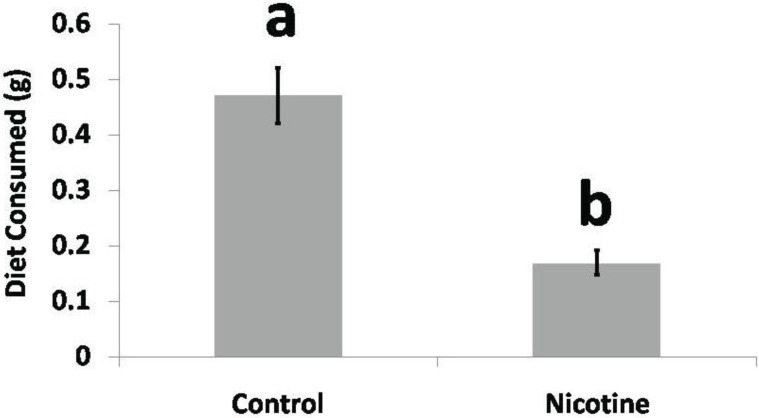
Diet preference bioassay. 6th instar *H. zea* caterpillars consumed significantly more artificial control diet than artificial diet laced with nicotine (1.5 µg nicotine/mg diet) over a 24 h observation period (N = 40, *t*-test, *p* = 8.8 × 10^−7^).

When given a choice of diets, caterpillars consumed more artificial diet than artificial diet laced with nicotine by a factor of 2.67 (Figure 2) (N = 40, *t*-test, *p* < 0.05).

#### 3.2.2. Observed Incidences of Cannibalism

**Figure 3:**
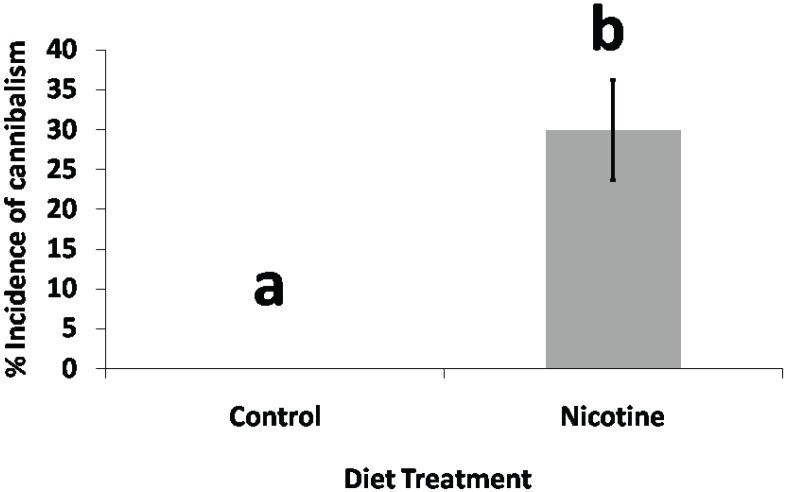
Cannibalism bioassay. *Helicoverpa zea* caterpillar pairs sharing artificial diet laced with nicotine (1.5 µg nicotine/ mg diet) encountered significantly more incidences of cannibalism than *H. zea* caterpillar pairs sharing artificial control diet (N = 60, Wilcoxon, *p* < 0.0044, df = 1).

We observed no cases of cannibalism among caterpillar pairs sharing artificial diet and 13 cases of cannibalism among caterpillar pairs sharing artificial diet laced with nicotine (Figure 3) (N = 60, Wilcoxon, *p* = 0.0044).

The phenomenon of increased incidence of cannibalism when *H. zea* caterpillars are confronted with a choice between nicotine and a conspecific caterpillar can be interpreted in a variety of ways. Bernays [5] postulates that polyphagous caterpillars supplement their diet or compensate for toxins in their environment by engaging in cannibalism. As an antifeedant, nicotine might indirectly compel caterpillars to prey upon one another by inducing starvation. As a potent neurotoxin, nicotine might also present a proximate cause for aggression in *H. zea* caterpillars. However, although possible relationships between nicotine and hostile behavior have not been explored in insects, nicotine has been documented to depress hostile tendencies in humans [25]. Previous studies on humans have identified a link between the neurotransmitter monoamine oxidase A (MAOA) and aggressive behavior [26].

Resource competition for space and resources has been suggested as a further possible reason for cannibalism in insects [8]. However, our observation that no incidences of cannibalism occurred among caterpillars feeding on control diet does not support resource competition as a possible cause for conspecific predation. From the perspective of ecological risk, the threat to fitness posed by the toxicity of nicotine might be balanced against the threat to fitness posed by attacking a conspecific. One model of foraging behavior in insects, for instance, has sought to define the toxicity of substances encountered by insects in terms of time required to neutralize or circumvent a plant’s defenses [27]. Although there is no evidence that caterpillars directly weigh the risks associated with their behavior, there have been several documented instances of insects attenuating their behavior in response to environmental stimuli from various trophic levels [28,29].

### 3.3. Overall Transcriptional Profile of H. zea

**Figure 4:**
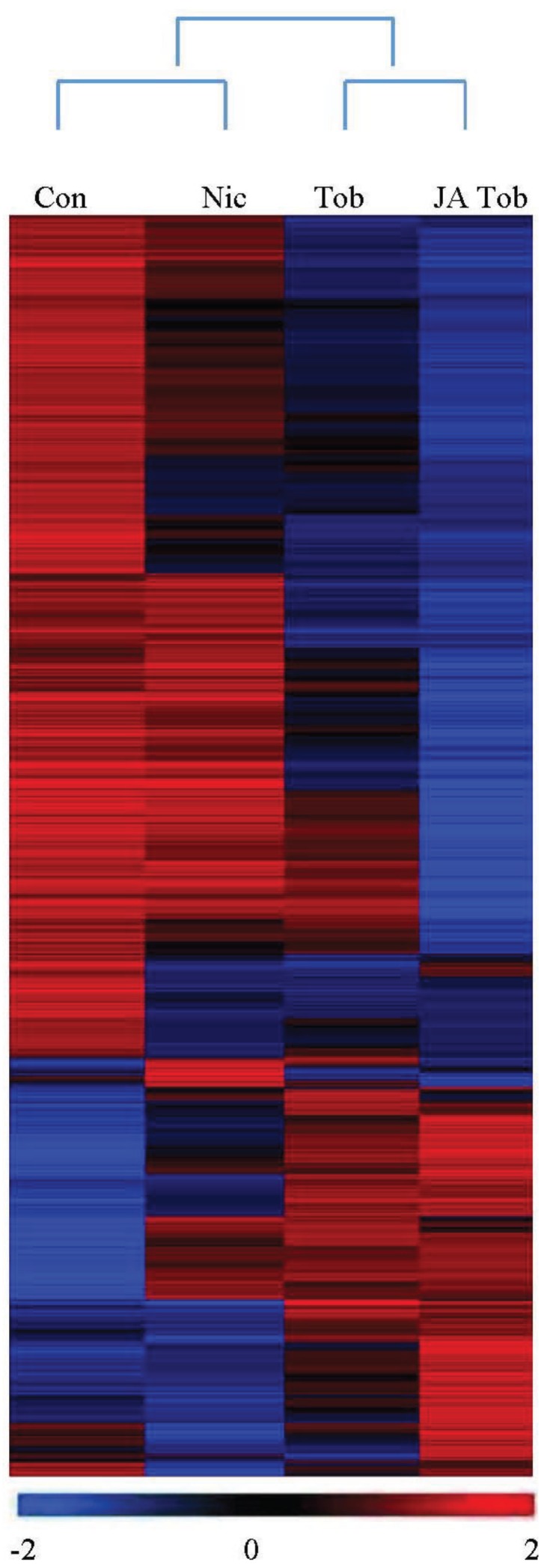
Cluster analysis of caterpillar genes up- and downregulated in response to artificial diet treated with nicotine (Nic), feeding on tobacco plants (Tob) or tobacco plants treated with jasmonate (JA Tob). The dendrogram on top of the clustered heat map illustrates the relationship among the four treatments. Genes upregulated by treatment appear in red (positive log2-ratio values), while those that were downregulated appear in blue (negative log2-ratio values). Each row in the column corresponds to mean of individual genes, and a color scale is presented below the figure.

Extracted RNA for *H. zea* caterpillars feeding on artificial diet, artificial diet laced with nicotine, *N. tabacum* (tobacco) plants, and tobacco plants treated with jasmonate were hybridized to microarray chips for each treatment. The transcriptome analysis revealed significant regulation of 2265 genes (one-way ANOVA, Benjamini and Hochberg *p* < 0.05) across the four treatments. The cluster analysis revealed an expected relationship among the four treatments where caterpillar genes stimulated by feeding on tobacco plants or jasmonate-treated tobacco plants were more similar to each other than feeding on artificial diet, or artificial diet treated with nicotine (Figure 4).

In general, more genes were suppressed by feeding on tobacco plants than the artificial diet, and even more so for caterpillars that fed on jasmonate-treated tobacco. Likewise, more genes were suppressed for caterpillars that fed on artificial diet treated with nicotine than the control artificial diet, but not to the same degree as caterpillars that fed on tobacco plants. However, despite a substantial number of genes suppressed, there were at least ~25% of the genes that were highly stimulated in caterpillars that fed on tobacco plants and generally more so for jasmonate-treated tobacco plants. The biochemical differences between the artificial diet coated with nicotine *versus* the tobacco plants is substantial. The differences beyond nicotine can be attributed to a range of the tobacco plant’s anti-nutritive defenses and secondary metabolites. Thus, transcriptomic profiles of caterpillars on plants would be strikingly different than on artificial diet, and the caterpillars’ response to tobacco has more similarities to caterpillar responses to other plants [7,20,30]. Utilizing the artificial diet experiment allowed precise examination of *H. zea* larval response specifically to nicotine.

The expression of caterpillar genes that were particularly stimulated by tobacco plants and jasmonate-treated tobacco plants included digestive genes such as proteases, lipases and as well as chitin and cuticle related structures (Table 2). Additional categories of gene expression affected represented a broad distribution of functional categories and, as before, a generally greater magnitude of alterations for caterpillar that fed on tobacco plants, and tobacco plants treated with jasmonate than the control diet treated with nicotine. The genes were sorted into functional categories pertaining to genes of interest such as carbohydrate, immune, and hormone related genes (Table 3) as well as chemosensory, cytochrome p450, and glutathione S-transferase (GST) related genes (Table 4). A supplementary file includes complete lists of the genes that were significantly altered (Supplementary File S1).

**Table 2:** Expression of proteases, lipases, and chitin and cuticle related genes. Gene name, fold change and NCBI reference sequence are indicated.

Probability		Fold Difference				Gene Description	
**Protease**							
***p* value**	***Q* value**	**Con**	**Tob**	**Ja Tob**	**Nic**	**Gene ID**	**Annotation**
0.0003	0.02	1.00	11.30	10.31	6.83	Har_00002206	aminopeptidase N
0.0000	0.00	1.00	16.08	44.26	6.60	Har_00003693	carboxypeptidase B precursor
0.0002	0.01	1.00	3.51	4.21	3.27	Har_00011784	chymotrypsin-like protease C3
0.0011	0.03	1.00	2.38	4.11	2.99	Har_00108436	chymotrypsinogen
0.0008	0.03	1.00	1.87	3.37	1.57	Har_00024333	fat body aminopeptidase
0.0015	0.04	1.00	6.73	9.41	4.22	Har_00003913	putative trypsin precursor Hz17
0.0003	0.02	1.00	2.83	5.16	2.05	Har_00002324	serine protease 6
0.0006	0.02	1.00	0.14	0.38	0.16	Har_00001036	serpin 28
0.0017	0.04	1.00	0.17	0.09	0.38	Har_00083724	serpin 6
0.0013	0.04	1.00	12.59	15.09	7.33	Har_00003940	trypsin precursor Hz1
0.0008	0.03	1.00	5.29	8.06	4.89	Har_00088037	trypsin-like protease
**Lipase**							
***p* value**	***Q* value**	**Con**	**Tob**	**Ja Tob**	**Nic**	**Gene ID**	**Annotation**
0.0002	0.02	1.00	7.47	16.32	3.58	Har_00001844	inactive lipase
0.0000	0.00	1.00	39.75	46.26	8.15	Har_00004503	lipase
0.0001	0.01	1.00	11.61	20.64	4.46	Har_00033090	lipase
0.0001	0.01	1.00	6.74	15.89	3.09	Har_00001997	lipase-like protein
0.0000	0.01	1.00	6.68	3.97	9.56	Har_00099849	pancreatic lipase 2
0.0002	0.02	1.00	2.20	3.49	0.38	Har_00115947	pancreatic triacylglycerol lipase
**Chitin and cuticle related**							
***p* value**	***Q* value**	**Con**	**Tob**	**Ja Tob**	**Nic**	**Gene ID**	**Annotation**
0.0000	0.00	1.00	4.99	6.08	1.69	Har_00063917	chitin binding PM protein
0.0001	0.01	1.00	4.94	5.17	1.89	Har_00001913	chitin deacetylase
0.0003	0.02	1.00	3.50	59.68	1.02	Har_00043945	cuticle protein 1
0.0008	0.03	1.00	8.06	66.50	2.24	Har_00043942	cuticle protein 1
0.0003	0.02	1.00	2.35	4.10	1.15	Har_00041025	cuticular protein CPR54
0.0002	0.01	1.00	2.94	12.34	1.23	Har_00082622	pupal cuticle protein
0.0023	0.05	1.00	1.83	4.55	1.50	Har_00082621	pupal cuticle protein
0.0003	0.02	1.00	2.32	8.20	1.15	Har_00013205	putative cuticle protein

**Table 3:** Expression of carbohydrate, immune and hormone related genes. Gene name, fold change and NCBI reference sequence are indicated.

Probability		Fold Difference				Gene Description	
**Carbohydrate**							
***p* value**	***Q* value**	**Con**	**Tob**	**Ja Tob**	**Nic**	**Gene ID**	**Annotation**
0.0000	0.00	1.00	13.40	12.07	2.18	Har_00076114	alpha-amylase
0.0017	0.04	1.00	0.54	0.02	0.75	Har_00018505	beta-glucosidase
0.0020	0.04	1.00	6.40	0.11	2.89	Har_00104504	fructosidase
0.0014	0.04	1.00	4.54	3.16	2.08	Har_00073019	glucose oxidase-like enzyme
0.0000	0.01	1.00	6.31	5.16	2.42	Har_00118222	glucosidase
0.0008	0.03	1.00	0.57	0.17	1.23	Har_00022381	glucosidase 2 subunit beta
0.0025	0.05	1.00	0.59	0.39	1.08	Har_00053711	malate dehydrogenase
0.0004	0.02	1.00	0.46	0.28	1.13	Har_00053712	malate dehydrogenase
0.0021	0.05	1.00	1.77	0.42	3.94	Har_00016370	fructose 1,6-bisphosphate aldolase
**Immune**							
***p* value**	***Q* value**	**Con**	**Tob**	**Ja Tob**	**Nic**	**Gene ID**	**Annotation**
0.0022	0.05	1.00	2.46	4.81	2.71	Har_00040030	antibacterial protein
0.0004	0.02	1.00	7.80	5.69	2.50	Har_00018468	azurocidin-like precursor protein
0.0011	0.03	1.00	0.15	0.16	0.22	Har_00063138	immunolectin-A precursor
0.0001	0.01	1.00	16.69	22.48	5.85	Har_00001788	insect intestinal mucin 4
0.0000	0.01	1.00	0.50	0.20	0.83	Har_00032536	leucine-rich repeat-containing protein
0.0000	0.01	1.00	0.37	0.11	0.52	Har_00002593	lysozyme
0.0000	0.01	1.00	3.65	5.33	1.54	Har_00090503	polycalin
**Hormones**							
***p* value**	***Q* value**	**Con**	**Tob**	**Ja Tob**	**Nic**	**Gene ID**	**Annotation**
0.0017	0.04	1.00	0.59	0.32	0.80	Har_00013687	13-dehydrecdysone 3b-reductase
0.0002	0.02	1.00	0.50	0.80	4.04	Har_00076173	13-dehydroecdysone 3alpha-reductase
0.0013	0.04	1.00	0.05	0.03	0.73	Har_00120046	basic juvenile hormone sensitive hemolymph protein
0.0009	0.03	1.00	0.18	0.05	0.86	Har_00040042	basic juvenile hormone-suppressible protein 1
0.0004	0.02	1.00	3.67	2.99	2.44	Har_00101526	carboxylesterase
0.0001	0.01	1.00	0.38	0.19	0.95	Har_00083637	cytosolic juvenile hormone binding protein 36 kDa
0.0001	0.01	1.00	5.10	16.75	2.21	Har_00121616	juvenile hormone binding protein
0.0007	0.03	1.00	24.93	33.73	10.02	Har_00118352	juvenile hormone epoxide hydrolase
0.0004	0.02	1.00	0.39	0.03	0.67	Har_00060924	juvenile hormone resistance protein I

**Table 4:** Expression of chemosensory proteins, cytochrome p450 and GST related genes. Gene name, fold change and NCBI reference sequence are indicated.

Probability		Fold Difference				Gene Description	
**Chemosensory Proteins**							
***p* value**	***Q* value**	**Con**	**Tob**	**Ja Tob**	**Nic**	**Gene ID**	**Annotation**
0.0020	0.04	1.00	7.73	11.13	5.20	Har_00070438	antennal binding protein
0.0004	0.02	1.00	0.70	0.39	0.82	Har_00044567	chemosensory protein
0.0000	0.00	1.00	0.72	0.31	0.70	Har_00079309	chemosensory protein 3
0.0002	0.01	1.00	0.01	0.09	0.05	Har_00046706	chemosensory protein 9
0.0018	0.04	1.00	0.62	0.12	0.95	Har_00030030	chemosensory protein CSP1
0.0003	0.02	1.00	0.87	0.24	0.83	Har_00004391	chemosensory protein CSP2
0.0024	0.05	1.00	0.37	0.34	0.85	Har_00051836	chemosensory-like protein
0.0002	0.01	1.00	0.45	0.27	0.64	Har_00053566	nicotinic acetylcholine receptor alpha 9
0.0002	0.01	1.00	15.19	26.94	7.54	Har_00039993	odorant-binding protein-2 precursor
0.0018	0.04	1.00	2.90	14.68	0.80	Har_00063353	sensory appendage protein-like protein
**Cytochrome p450**							
***p* value**	***Q* value**	**Con**	**Tob**	**Ja Tob**	**Nic**	**Gene ID**	**Annotation**
0.0009	0.03	1.00	0.33	0.52	0.20	Har_00000415	antennal cytochrome P450 CYP9
0.0013	0.04	1.00	0.08	0.07	0.06	Har_00002998	cytochrome P450
0.0010	0.03	1.00	1.75	1.68	8.64	Har_00003806	cytochrome P450 6B27
0.0005	0.02	1.00	0.53	0.10	2.69	Har_00000851	cytochrome p450 CYP337B1
0.0015	0.04	1.00	0.28	0.17	0.78	Har_00000866	cytochrome P450 CYP4M7
0.0007	0.03	1.00	0.58	0.29	0.65	Har_00000524	gossypol-induced cytochrome P450
0.0023	0.05	1.00	0.59	0.25	3.29	Har_00024682	heat shock protein 20.1
0.0009	0.03	1.00	0.57	0.30	0.39	Har_00024281	heat shock protein 90
0.0004	0.02	1.00	0.47	0.18	0.79	Har_00077695	HSP90 cochaperone CDC37
**GST**							
***p* value**	***Q* value**	**Con**	**Tob**	**Ja Tob**	**Nic**	**Gene ID**	**Annotation**
0.0009	0.03	1.00	0.38	0.33	0.68	Har_00026463	glutathione S-transferase
0.0021	0.05	1.00	3.17	13.37	2.43	Har_00002152	glutathione S-transferase GSTX01
0.0004	0.02	1.00	0.45	0.46	0.75	Har_00058848	glutathione S-transferase omega 1

### 3.4. Protein and Lipid Digestion

**Figure 5:**
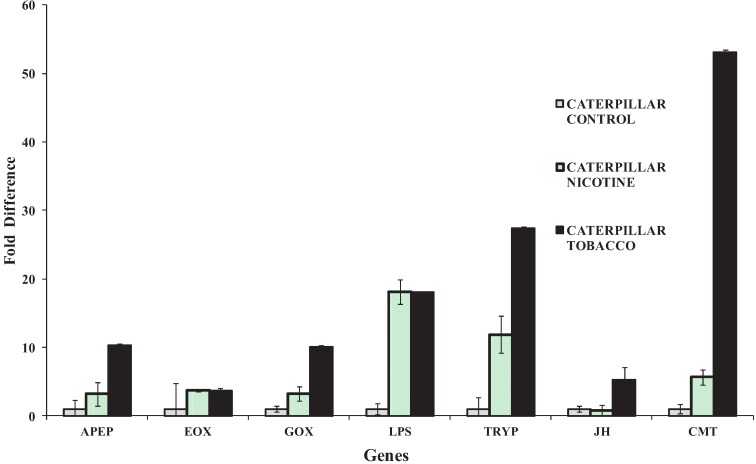
Relative fold changes (y-axis) of gene expression of caterpillars that fed on artificial diet (control) compared to caterpillars that fed on artificial diet treated with nicotine (nicotine) and caterpillars that fed on tobacco plants (tobacco). The altered expression of the genes of interest include aminopeptidase (APEP), ecdysone oxidase (EOX), glucose oxidase (GOX), lipase (LPS), trypsin (TRYP), juvenile hormone inducible protein (JH) and chymotrypsin (CMT). Bars indicate means ± Standard Error and *N* = 4 biological replicates. Different letters indicate significance with ANOVA and Fisher’s LSD at *p* < 0.05.

Our observation that incidences of cannibalism (Figure 3) increase with exposure to nicotine agrees with Bernays’ [5] suggestion that polyphagous caterpillars supplement their diet or compensate for food source toxicity by feeding on one another. At the same time, our microarray experiment exhibits a prominent stimulation of transcripts related to various proteases and lipases (Table 2; Figure 5). The list of genes upregulated in response to diets of nicotine and tobacco include proteases such aminopeptidase N, carboxypeptidase, chymotrypsin, and trypsin precursors and several lipases including pancreatic triacylglycerol lipases (Table 2); these enzymes exemplify the observed increase in protease and lipase transcription. Although the function of these proteases can be generalized as aiding in protein digestion by cleaving peptide bonds in amino acid chains, the abundance and diversity of proteases present in lepidopteran midguts is thought to indicate a close correspondence with specific, possibly defense-related, proteins produced by plants [31]. Moreover, the simultaneous downregulation of transcription for genes encoding a variety of serpin protease inhibitors (Table 2) further suggests that *H. zea* caterpillars compensate for plant anti-nutritive defenses by maximizing protein intake or possible functions as a regulatory protein.

### 3.5. Chitin Binding

Chitin and cuticle related genes were substantially stimulated on tobacco plants and substantially more for jasmonate-treated plants while not particularly altered even for the nicotine-laced diet. Chitin binding peritrophic membrane protein, chitin deacetylase, and a variety of cuticular proteins were all stimulated (Table 2), including the insect intestinal mucin gene noted above. The lepidopteran peritrophic matrix forms an important barrier to physical damage caused by the abrasion of plant cell walls and structures as well as crystalline toxins, various anti-nutritive enzymes, and pathogenic infections [32]. Remodeling of the peritrophic matrix by changing the composition of cuticle and chitin-binding proteins could lead to increased resistance against physical damage or the ingested plant defensive proteins and secondary metabolites.

### 3.6. Carbohydrate Metabolism

Although there is some overlap in gene regulation between the experimental treatments, several trends in transcription were more highly altered in the tobacco treatment. For example, the expression of glucosidases was generally variable. In one instance, one of the glucosidases was downregulated for tobacco and even more so for jasmonate-treated tobacco, but to a lesser extent by the nicotine-laced diet. Meanwhile, another glucosidase was more stimulated in the tobacco plant than it was in the nicotine-laced diet (Table 3) [33]. The expression of alpha-amylase, an enzyme responsible for degrading starch, was more pronounced in the tobacco treatment than the nicotine-laced diet (Table 3) [34]. Fructosidase expression was stimulated for tobacco plants and nicotine-laced diet, but was suppressed for jasmonate-treated tobacco. These results reflect that the expression of genes in *H. zea* could be responsive to the differences in food source composition as well as to tobacco anti-nutritive defenses which could result in the differential regulation of specific enzymes in the insect midgut.

Glucose oxidase (GOX), a salivary enzyme, was upregulated in response to tobacco, and to a lesser degree to the nicotine-laced diet (Figure 5; Table 3). Glucose oxidase is the *H. zea* caterpillar salivary factor responsible for suppression of nicotine-induction in tobacco plants and increased survival of larvae [35,36]. Although GOX is well-documented in *H. zea* as an adapted defense to the induction of nicotine, the factors responsible for upregulating GOX in caterpillars have not yet been identified. Furthermore, alteration of the caterpillar enzyme can lead to changes in the transcriptomic response of the plants upon which the caterpillar feeds [19,30].

### 3.7. Immune Genes

Several genes associated with insect immune response were both stimulated and suppressed in the experimental treatments (Table 3). While we have no reason to suspect that the *H. zea* subjects in our experiments were differentially exposed to substantial pathogens; these agents would certainly exist in the environment to some degree. However, none of the caterpillars or plants showed any overt signs of infection. In addition, the plants were grown in a clean growth chamber on commercial medium. If the caterpillars carried pathogens, then all caterpillars would be expected to carry similar pathogens as they were obtained from the same source and treated the same during each replicate and experiment. Yet, genes coding for an antibacterial protein, an azurocidin-like precursor protein, insect intestinal mucin, and polycalin (Table 3), were upregulated during the course of the experiments, particularly for the tobacco plant treatments. Since it has been demonstrated that even simple oral uptake of bacteria can induce immune-related genes in lepidopteran herbivores, the upregulation of these putative immune-related genes in the plant feeding experiments could relate to the occurrence of bacteria associated with the ingested plant material [32]. Azurocidin has been identified as an anti-microbial agent associated with insect immunity, while mucins in insects are secreted in the peritrophic matrix, where they serve to apprehend pathogens and their toxins [32]. However, lysozyme, an anti-microbial agent commonly secreted in hemolymph and insect saliva [37], was downregulated. Noland *et al.* [16] determined that lysozyme was stimulated in *H. zea* caterpillars infected with *Helicoverpa zea* single nucleopolyhedrovirus (HzSNPV). Regardless, under field conditions, exposure to potential pathogens would be a frequent occurrence for *H. zea*; this immune defense may be triggered by some unknown plant factors or simply elevated levels of bacteria found on non-sterile plant material.

### 3.8. Hormones and Growth

Our results indicate that the growth rates of *H. zea* caterpillars are reduced in response to the presence of nicotine in their food source. However, our observation that several growth-related genes are differentially transcribed raises the question of whether the overall change in growth rate represents an adaptive response to nicotine or a symptom of toxicity. From an adaptive perspective, the observed pattern in gene regulation could be a physiological adjustment to the ingestion of nicotine and tobacco, whereby *H. zea* caterpillars curtail their growth rates to permit slower feeding rates on toxic substances. Alternatively, the suppressed expression of these growth-related genes could be a symptom of nicotine toxicity; by prolonging caterpillar maturation, tobacco plants would benefit by providing potential predators of *H. zea* caterpillars with more time to spot and attack their prey.

If the reduced growth rates observed in our bioassay (Figure 1) were to be attributed exclusively to the toxicity of nicotine, only specific aspects of *H. zea* physiology might be expected to be targeted. However, the microarray data reveal that growth-related genes are differentially transcribed in a broad distribution across the transcription of *H. zea* caterpillars feeding on both nicotine-laced diet and tobacco. In the endocrine system, 13-dehydroecdysone 3-alpha-reductase, a precursor to ecdysone, the hormone responsible for regulating insect molting, is downregulated (Table 3) [38]. Another significantly stimulated gene related to the endocrine system, juvenile hormone epoxide hydrolase, is known to play a central role in modulating metamorphosis [39] (Table 3).

### 3.9. Chemosensory

Diets rich in carbohydrates can mask the taste of substances that would otherwise be noxious to caterpillars [40,41], and electrophysiological experiments have demonstrated that taste receptor neurons in the sensory maxilla of *H. armigera* caterpillars accustomed to feeding on strychnine and stropanthine-K display reduced sensitivity to the deterrent chemicals, accompanied by a lack of aversive behavior [42]. Therefore, we conducted the first bioassay experiment (Figure 1) to establish that the *H. zea* caterpillars used in the experiments could detect and react to the presence of nicotine in the provided nutrient-rich artificial diet. The observed aversive behavior of *H. zea* caterpillars towards nicotine-laced diet agrees with previous research demonstrating that the bitter taste associated with many alkaloids, including nicotine, acts as a feeding deterrent [41].

The microarray data show that the sensory-related transcripts coding for odorant-binding protein-2 precursor, antennal binding protein and sensory appendage protein were all highly stimulated in response to feeding on tobacco plants and to a lesser degree to the nicotine-laced diet (Table 4).This could be due to compounds produced in whole tobacco plants especially the jasmonate-treated plants. However, the expression of chemosensory-like proteins and the nicotinic acetylcholine receptor alpha 9 subunit gene was downregulated in response to both nicotine-laced diet and tobacco.

The observed differential regulation of genes associated with chemical perception can be interpreted in numerous ways. In their essential role as binders of small lipophilic molecules, odorant binding and chemosensory proteins (OBPs and CSPs) are present in tissues throughout the organism [43,44]. Beyond binding substances present in the environment, OBPs and CSPs also shuttle small lipophilic molecules, such as developmental hormones, between tissues not directly associated with perception within the organism (for review, see Pelosi *et al.* [45]).

Although the data do not show a direct link between all the listed sensory-related genes and the observed aversion to nicotine, genes coding for sensory proteins and neural receptors are traditional candidates in gene-to-behavior studies as they mark a change in the way an organism perceives its environment (for reviews, see [46,47]). In a number of a species, proteins associated with chemical perception enable pheromone detection and, more generally, lipophilic substances in the environment [48]. As such, our results indicate that odorant-binding protein-2 precursor and antennal binding protein may be involved in the observed aversive behavior of *H. zea* caterpillars towards nicotine-laced diet.

Of the genes identified by the microarray analysis, nicotinic acetylcholine receptor alpha 9 subunit (Table 3) is the most suspect candidate for modifying caterpillar behavior, as no other plausible neurotransmitters were significantly transcribed in the dataset. Nicotinic acetylcholine receptors (nAChRs) have been extensively studied in both vertebrate and invertebrate systems for the many roles they play in modulating cognition by mediating cholinergenic signaling between synapses (for vertebrate reviews see [48]; in insects see [49,50,51]). Nicotine as well as the neonicotinoid class of synthetic pesticides target the insect nervous system by acting either as agonists or antagonists with nAChR binding sites [48]. While several nAChRs have been identified in the silkworm *Bombyx mori* genome, no studies to date have addressed their role in the behavior of lepidopteran larvae [51]. NAChRs mediate odor-associated learning in the fruit-fly *Drosophila melanogaster* [52]. Similarly, inhibition of nAChRs in honey bees, *Apis mellifera*, leads to decreased rates of learning and long-term memory acquisition [50]. Although our study does not identify a specific role for the differential transcription of nAChR alpha subunit 9, the previously described role for odor-associated learning in *D. melanogaster* and *A. mellifera*, taken into consideration with the expression of chemosensory genes observed in our microarray data, would suggest that nAChR subunit 9 is associated with the observed aversive and cannibalistic behavior of *H. zea* caterpillars to their environment. Alternatively, given the role that they perform in memory and learning in other insect species, it is possible that the action of nAChRs form a long-term memory in caterpillars. Recent evidence demonstrated that the experiences of Lepidoptera as caterpillars are retained into adulthood, where they might influence host-plant selection during oviposition [53]. As a third possibility, nAChR subunit 9 as well as other sensory genes could be downregulated in response to nicotine to decrease sensitivity to the toxin.

### 3.10. Detoxification

The cytochrome P450 enzymes comprise a large enzyme superfamily including a broad class of detoxicants that serve as a mainstay in insect countermeasures against plant allelochemicals [54] along with glutathione S-transferase genes that are responsible for intracellular detoxification and aid in the resistance of insecticides [55,56] (Table 4). In the *Nicotiana* specialist caterpillar *M. sexta*, cytochrome P450 enzymes are responsible for conferring nicotine-tolerance, likely by metabolizing ingested nicotine before it can bind to neuroreceptors [24]. In specialist species, some cytochrome P450s have a narrower substrate binding affinity than their counterpart enzymes present in generalist species [32]. Accordingly, our data reflect that *H. zea* caterpillars differentially regulate the expression of several cytochrome P450 and glutathione S-transferase genes in each experimental treatment. Rather than uniform regulation, some of the P450 enzymes are downregulated while others are upregulated (Table 4).

The simultaneous action of multiple cytochrome P450 enzymes metabolizing several allelochemicals has at times been known to yield metabolites that are more toxic to the organism than the originating substrate [54]. Hence, selectively inhibiting or downregulating some cytochrome P450s is thought to reduce the emergence of toxic degradation products [54]. This effect may explain the simultaneous up- and downregulated of cytochrome P450 enzymes evidenced in our microarray data (Table 4). Furthermore, the upregulated cytochrome P450s identified in the generalist herbivore *H. zea* are prime candidates for testing efficacy in converting nicotine to less toxic metabolites.

### 3.11. Real Time Quantitative Polymerase Chain Reaction

Several genes of interest were analyzed by qPCR in order to validate the caterpillar’s response to feeding on artificial diet laced with nicotine or tobacco plants compared to the artificial control diet. The data confirm transcriptional stimulation of genes of interest such as aminopeptidase (APEP), ecdysone oxidase (EOX), glucose oxidase (GOX), lipase (LPS), trypsin (TRYP), juvenile hormone inducible protein (JH) and chymotrypsin (CMT). Transcription of these genes was generally more pronounced in caterpillars feeding on tobacco plants and to lesser extent for caterpillars that fed on nicotine-treated diet in comparison to the control (Figure 5).

## 4. Conclusions

The most pronounced trends in gene transcription reflected by our microarray analysis are the broad regulation of proteases and lipases, as well as genes related to chitin, hormones, pathogen and detoxification related genes. These results are in agreement with the body of previously published literature in plant–herbivore interactions [6,56]. However, the combination and diversity of proteases and cytochrome P450s simultaneously expressed reflect a complexity of interaction between plants and herbivores that could not be fully appreciated without recent advances in molecular techniques (for discussion, see [7,19,57]). Likewise, we observed that behavioral changes in *H. zea* in response to dietary nicotine are accompanied by a suite of changes in genes likely related to chemoreception.

Aside from motifs in gene expression, the microarray data frame an ambiguity as to whether the observed changes in growth, behavior and transcription signify an adaptive response to plant defenses on the part of the caterpillar, or that they instead signify a consequence of toxicity on the part of the plant. Characterizing *H. zea*’s response to the toxicity of nicotine and tobacco as compensatory is convenient because it avoids implying a particular causal relationship between plant and herbivore. Instead, the term emphasizes the close correspondence between the physiology of herbivore and plant.

## Supplementary Files

Supplementary File 1
